# A Label‐Free Hyperspectral Imaging Device for Ex Vivo Characterization and Grading of Meningioma Tissues

**DOI:** 10.1002/jbio.202500374

**Published:** 2025-12-15

**Authors:** Pietro Ricci, Camilla Bonaudo, Ivan Ezhov, Anam Toaha, Dorotea Nardini, Manuel Camelia, Federica Lucidi, Filippo Nozzoli, Tim Mach, Ilias Tachtsidis, Daniel Rueckert, Alessandro Della Puppa, Luca Giannoni, Francesco S. Pavone

**Affiliations:** ^1^ Department of Physics and Astronomy University of Florence Sesto Fiorentino Italy; ^2^ European Laboratory for Non‐Linear Spectroscopy Sesto Fiorentino Italy; ^3^ Neurosurgery, Department of Neuroscience Psychology, Pharmacology and Child Health, University of Florence, Azienda Ospedaliero‐Universitaria Careggi Florence Italy; ^4^ Klinikum Rechts der Isar Technischen Universität München Munich Germany; ^5^ Histopathology and Molecular Diagnostics Careggi University Hospital Florence Italy; ^6^ Department of Medical Physics and Biomedical Engineering University College London London UK; ^7^ Department of Computing Imperial College London London UK; ^8^ Munich Center for Machine Learning (MCML) Munich Germany; ^9^ National Institute of Optics, National Research Council Sesto Fiorentino Italy

**Keywords:** Hyperspectral Imaging, Meningiomas, Tumor Grade Discrimination

## Abstract

Histopathology remains the gold standard for definitive tumor diagnosis after surgical resection; however, its lengthy processing time can delay critical postoperative care. Hyperspectral imaging (HSI) is emerging as a promising label‐free technique for rapid biochemical tissue assessment. Here, we present HyperProbe1.1 (HP1.1), an HSI system designed for noninvasive analysis of fresh brain tumor biopsies. In this proof‐of‐concept study, we applied the HP1.1 system to freshly excised meningioma specimens—the most common primary intracranial tumors. The platform enabled rapid, label‐free mapping of metabolic activity and vascular heterogeneity, while spectral unmixing further allowed the quantification of endogenous biomarkers such as cytochrome c oxidase (CCO), hemoglobin derivatives, and lipids, revealing molecular patterns consistent with histopathological tumor grading according to the 2021 WHO classification. These results highlight the feasibility of HSI for rapid biochemical tissue assessment and its potential integration into intraoperative decision‐making.

## Graphical abstract

HyperProbe1.1 enables rapid, label‐free biochemical mapping of freshly resected meningiomas. By quantifying endogenous biomarkers such as cytochrome c oxidase, hemoglobin derivatives, and lipids, the system reveals molecular signatures consistent with tumor grading and generates spatial maps that visualize metabolic and vascular heterogeneity across the tissue.

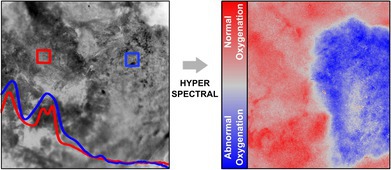

## 1 Introduction

In neurosurgical oncology, a rapid and reliable histopathologic diagnosis plays a critical role in guiding postoperative treatment [1]. However, conventional histology requires fixation, processing, and staining steps that delay results by several days—an interval that can be critical for patients needing early adjuvant therapies such as radiotherapy or chemotherapy. In the acute postoperative setting, treatment planning generally proceeds without definitive tumor grading, which can affect therapeutic decisions, prognosis prediction, and patient counseling. This limitation underscores the need for diagnostic tools capable of providing rapid intraoperative or perioperative information on tumor type and grade, ideally with real‐time functional contrast to support intraoperative decision‐making and neuronavigation.

Among brain tumors, meningiomas stand out for their high prevalence and well‐characterized pathology, making them a suitable model for developing rapid diagnostic and grading approaches. They are the most common primary intracranial neoplasms, accounting for approximately 38% of all central nervous system tumors and more than half of benign cases [2]. They occur from arachnoid cap cells and are typically slow‐growing, benign, and noninfiltrating neoplasms. The 2021 World Health Organization (WHO) classification categorizes meningiomas into three grades based on histopathological and molecular features [3]: grade 1 (≈80% of cases) shows low proliferative activity and well‐defined borders; grade 2 (15%–20%) exhibits increased mitotic activity and a higher recurrence rate; grade 3 (1%–3%) is rare but highly aggressive, with rapid growth, cellular atypia, and frequent relapse [4].

Surgical resection remains the cornerstone of both diagnosis and treatment, with the Simpson grading system describing the extent of tumor removal [5]. Accurate grading is therefore essential, as it directly influences the surgical strategy, the indication for adjuvant therapy (e.g., GammaKnife [6, 7]), and the design of follow‐up protocols. Yet, no intraoperative technique currently provides real‐time histological grading, leaving neurosurgeons to rely on anatomical landmarks and preoperative imaging [8]—approaches often insufficient for precise tissue characterization.

Recent optical methods, such as Raman and fluorescence spectroscopy, have been explored to bridge this gap by enabling rapid, label‐free tumor identification [9]. Although compact and potentially intraoperative, these point‐based approaches sample limited tissue areas and require mechanical scanning to assess spatial heterogeneity.

Hyperspectral imaging (HSI) has emerged as a powerful alternative, capturing full‐field molecular and structural information simultaneously [10, 11, 12, 13]. This contact‐free, label‐free modality provides rapid biochemical mapping of fresh tissue, offering a new dimension of optical contrast that could complement histopathology [14].

To address this need, we recently developed HyperProbe1.1 (HP1.1) [15, 16], a compact and transportable HSI system designed for the analysis of fresh ex vivo brain tumor specimens over centimeter‐scale fields of view (FOVs). The previous version (HP1.0) demonstrated its ability to generate detailed biomolecular maps from fresh glioma biopsies without fixation or staining [17]. In this proof‐of‐concept study, we apply HP1.1 to ex vivo meningioma samples to explore whether hyperspectral data can reveal molecular and metabolic features associated with histopathological grading. By generating oxygenation and metabolic contrast maps that highlight tissue heterogeneity, HP1.1 could support intraoperative neuronavigation and contribute to faster, more informed clinical decisions in meningioma management.

## 2 Materials and Methods

### 2.1 HSI

**FIGURE 1:**
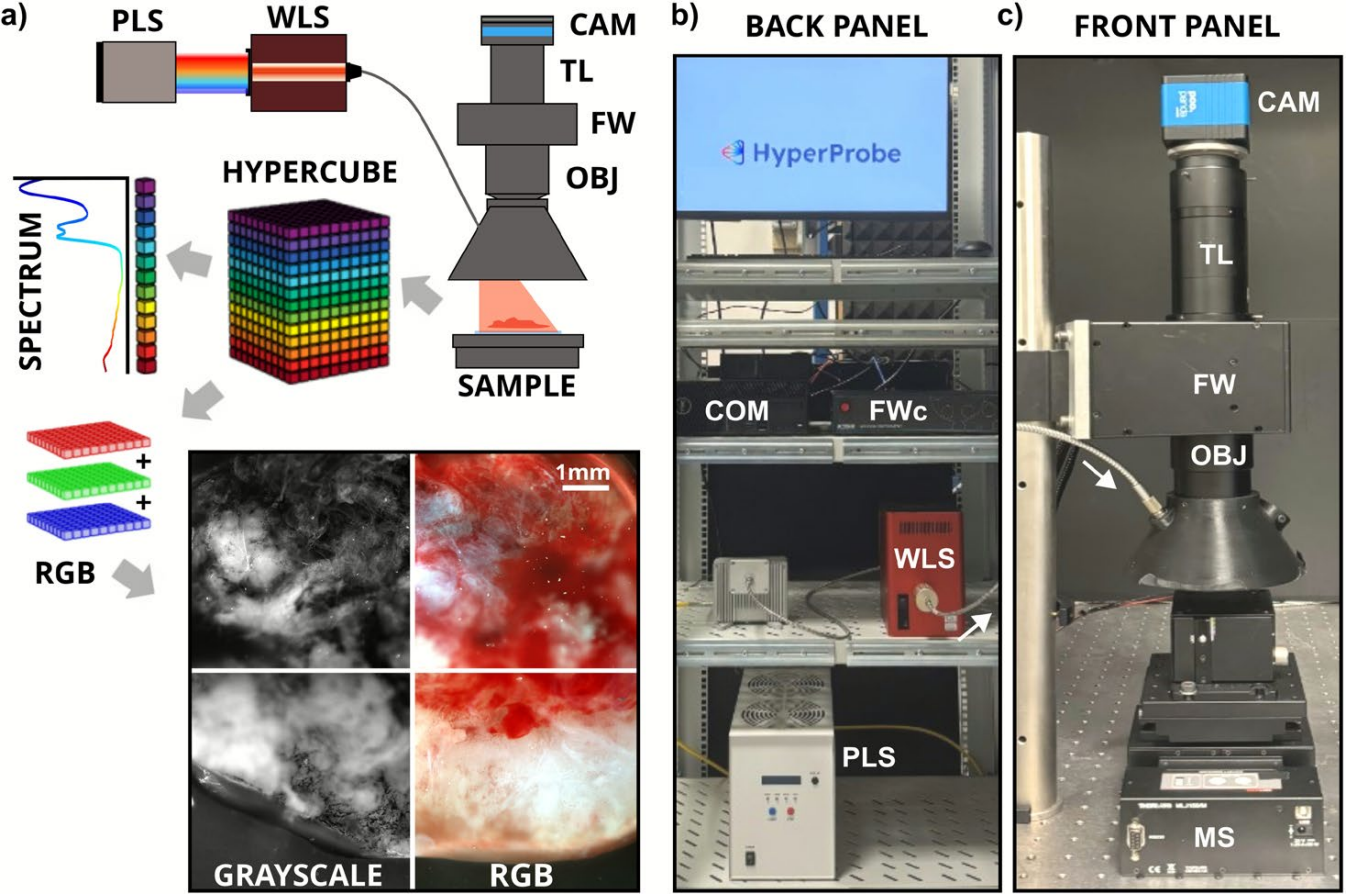
(a) Schematic of HyperProbe with all its components. In red, the light from the optical fiber is selected as an output example. The stacked grayscale images that compose the hypercube are colored for correspondence with the different illumination wavelengths. On the left, a column of pixels contains full spectral information. Red, blue and green layers are selected from the hypercube to reconstruct an RGB composite (right) over half of the original grayscale image (left); (b and c) Pictures of the back and front panel of HyperProbe, respectively. CAM, camera; COM, computer; FW, filter wheel; FWc, filter wheel controller; MS, motorized stage; OBJ, objective; PLS, plasma light source; TL, tube lens; WLS, wavelength selector.

The HSI setup—the HP1.1—employs a broadband plasma‐based light source (XWS‐65, Isteq), emitting across a spectral range from 250 to 2500 nm. Spectral selection is achieved via a flexible wavelength selector (FWS‐Poly‐CUS‐10, Spectrolight), capable of filtering the output between 385 and 1015 nm, with user‐defined bandwidths from 3 to 15 nm and sub‐200 ms switching times. Light is delivered through a 1‐mm core optical fiber (FC‐IR1000‐1, Avantes) and directed onto the sample surface. The light source is mounted on the inner surface of a custom black plastic cone designed to reduce ambient light interference during acquisition. An illumination angle of ~35° prevents the specular reflections from the sample and minimizes optical artifacts during hyperspectral acquisitions. Reflected light is collected using an apochromatic macro‐objective (SDF PLAPO 1X PF, Olympus) and focused by a tube lens onto a scientific CMOS camera (pco.panda 4.2, PCO), offering a resolution of 2048 × 2048 pixels, frame rates up to 40 fps, and high quantum efficiency in the visible and near‐infrared spectrum. A schematic of the optical path and photos of the back and front panels of the HP1.1 are shown in Figure 1a–c.

### 2.2 Hypercube Generation

Image stacks, or hypercubes, were generated by sequentially illuminating the sample at discrete wavelengths (Figure 1a). For system validation, the broadband source was spectrally tuned from 385 to 1015 nm in 5 nm increments, each with a bandwidth of 5 nm. Therefore, at each wavelength, narrowband illumination was projected onto the sample, and the reflected light was captured in widefield mode without additional filtering in the detection path. This procedure produced a hyperspectral cube composed of a series of 2D reflectance images, where each pixel contains the full spectral profile along the third dimension. This configuration allows pixel‐wise spectral analysis and facilitates the identification of biochemical or morphological features based on their reflectance or attenuation profiles.

By selecting the monochromatic images acquired illuminating the sample with blue (470 nm), green (530 nm) and red (650 nm) light, and properly combining them in postprocessing, it is possible to reconstruct an RGB composite version of the image, useful to highlight spectral contrast and reveal spatial patterns or structures that are not obvious in individual grayscale bands (Figure 1a, bottom).

### 2.3 Data Calibration and Processing

Before conducting experiments, the system underwent calibration to correct for spectral variations in illumination and detection. A white reference (Spectralon5″, nominal reflectance 99%, Labsphere) was imaged using constant integration time to evaluate the system response. These data were used to adjust the exposure time for each wavelength and to ensure uniform signal intensity. All subsequent sample acquisitions used this exposure calibration. During image acquisition, ambient light in the laboratory was turned off. For each sample measurement I(*x*, *y*, *λ*), two additional hypercubes were taken: *I*_white_(*x*, *y*, *λ*), from the white reference target, and *I*_dark_(*x*, *y*, *λ*), with all light sources off. The sample reflectance pixel *R*(*x*, *y*, *λ*) was calculated after dark subtraction and normalization to the reference:

(1) $$R\left(x,y,\lambda \right)=\frac{I\left(x,y,\lambda \right)-\frac{t_{s}}{t_{d}}\cdot I_{\text{dark}}\left(x,y,\lambda \right)}{\frac{t_{s}}{t_{w}}\cdot I_{\text{white}}\left(x,y,\lambda \right)-\frac{t_{s}}{t_{d}}\cdot I_{\text{dark}}\left(x,y,\lambda \right)}$$

Here, *t*_*s*_, *t*_*d*_, and *t*_*w*_ denote acquisition times for the sample, dark, and white measurements, respectively. Light attenuation *A*(*x*, *y*, *λ*), incorporating both absorption and scattering, was derived via:

(2) $$A\left(x,y,\lambda \right)=-\log_{10}R\left(x,y,\lambda \right)$$

### 2.4 Spectral Analysis

To infer molecular composition variations across samples, a rapid spectral unmixing method based on the modified Beer–Lambert Law (MBLL) was applied [18]. This method allows for the decomposition of a reflection spectrum into constituent components representing tissue molecular chromophores. For the meningioma biopsy analysis, we considered molecules such as water, fat, oxy‐ and deoxyhemoglobin (HbO_2_ and HHb), as well as reduced and oxidized forms of cytochrome (cyt‐b, cyt‐c, and cytochrome c oxidase (CCO)). Upon unmixing, the differences in molecular concentrations (with respect to a reference sample) are inferred for every pixel in an HSI biopsy image. We then compute an average concentration per image for every molecule of interest for the downstream analysis of tumor malignancy grading [18]. Image‐level, mean concentration values are reported for downstream exploratory comparisons across FOV. However, the unmixing procedure yields pixel‐wise concentration maps that preserve spatial structure and intratumor heterogeneity. To generate the inferred concentration maps, we focused on the spectral range between 500 and 900 nm, which captures the most relevant absorption features of HbO_2_, HHb, differential CCO (DiffCCO; the difference between oxidized and reduced CCO), and lipids. This wavelength window also allows adequate tissue penetration, making it suitable for reliable spectroscopic analysis. The estimated compositions—expressed either as concentrations or as volumetric contents—are reported in units of (mM/cm) and (cm^−1^).

### 2.5 Sample Preparation and Grading

Clinical, radiological, and intraoperative data were collected from patients who were operated on for suspected extra‐axial lesions at the Neurosurgical Department of the University Hospital of Careggi in Florence. All operations were performed by the same senior neurosurgeon (A.D.P.). Meningioma samples were obtained after routine neurosurgical operations, without any alteration of the diagnostic‐therapeutic pathway of patients, under ethical approval (Studio ID: 23672–23 672_BIO) and signature of patient‐informed consent.

Before acquisition, meningioma samples, typically between 0.5 and 2 cm in size, were rinsed in phosphate‐buffered saline (PBS) to remove surface contaminants. A thin coverslip was applied to flatten the sample and ensure even focus across the imaging field, besides preventing specular reflections from the tissue surface, residual fluids, or similar artifacts. Imaging was performed within 1 h of surgical removal. For larger specimens exceeding the system's FOV (0.65 × 0.65 cm^2^), multiple acquisitions were performed on separate regions to explore local heterogeneity, for a total of 43 FOVs from 21 analyzed samples.

The hematoxylin and eosin (H&E) stained slides and formalin‐fixed paraffin‐embedded (FFPE) tissue specimens, obtained by surgical resection, were extracted from the archive of the Pathology Unit, Careggi University Hospital (Florence, Italy). Tumor diagnosis and grading were assessed according to the 2021 WHO classification system.

## 3 Results

### 3.1 Multimodal Characterization of Meningioma Samples Through MRI, Histology, and HSI

Between October 2024 and April 2025, we collected clinical, radiological, and intraoperative data from 24 patients, three of whom were excluded because the acquisition quality was not adequate. The final cohort included 21 patients (M:F = 8:13, mean age 60.3 years): 14 grade 1 (eight transitional, one secretory, one psammomatous, one meningothelial, three fibrous), 6 grade 2 (four atypical, two choroid), and 1 grade 3 (anaplastic) meningioma (Table 1). Lesion locations were heterogeneous in the full cohort (15 supratentorial and 6 infratentorial). Among supratentorial lesions, three were right frontal, two parasagittal, and others involved the falx, clinoid process, olfactory groove, or temporal convexity; infratentorial lesions included foramen magnum, petroclival, and pontocerebellar meningiomas.

**TABLE 1:** Patient characteristics and dataset information.

Patients	Mean age	M:F	Grade 1	Grade 2	Grade 3	1 FOV	2 ≤ FOVs ≤ 3	> 3 FOVs
21	60.3	8:13	14	6	1	11	7	3

For larger specimens, multiple FOVs were imaged to characterize intratumor heterogeneity; however, these FOVs were not individually coregistered to distinct H&E sections in the present study. As a result, FOV‐level differences may reflect genuine spatial heterogeneity within the same histopathologic grade rather than incorrect grading. Indeed, even though the histopathological report provides a single categorical diagnosis per specimen and does not capture regional metabolic or vascular variability, the result represents the unquestioned gold standard for tumor classification.

**FIGURE 2:**
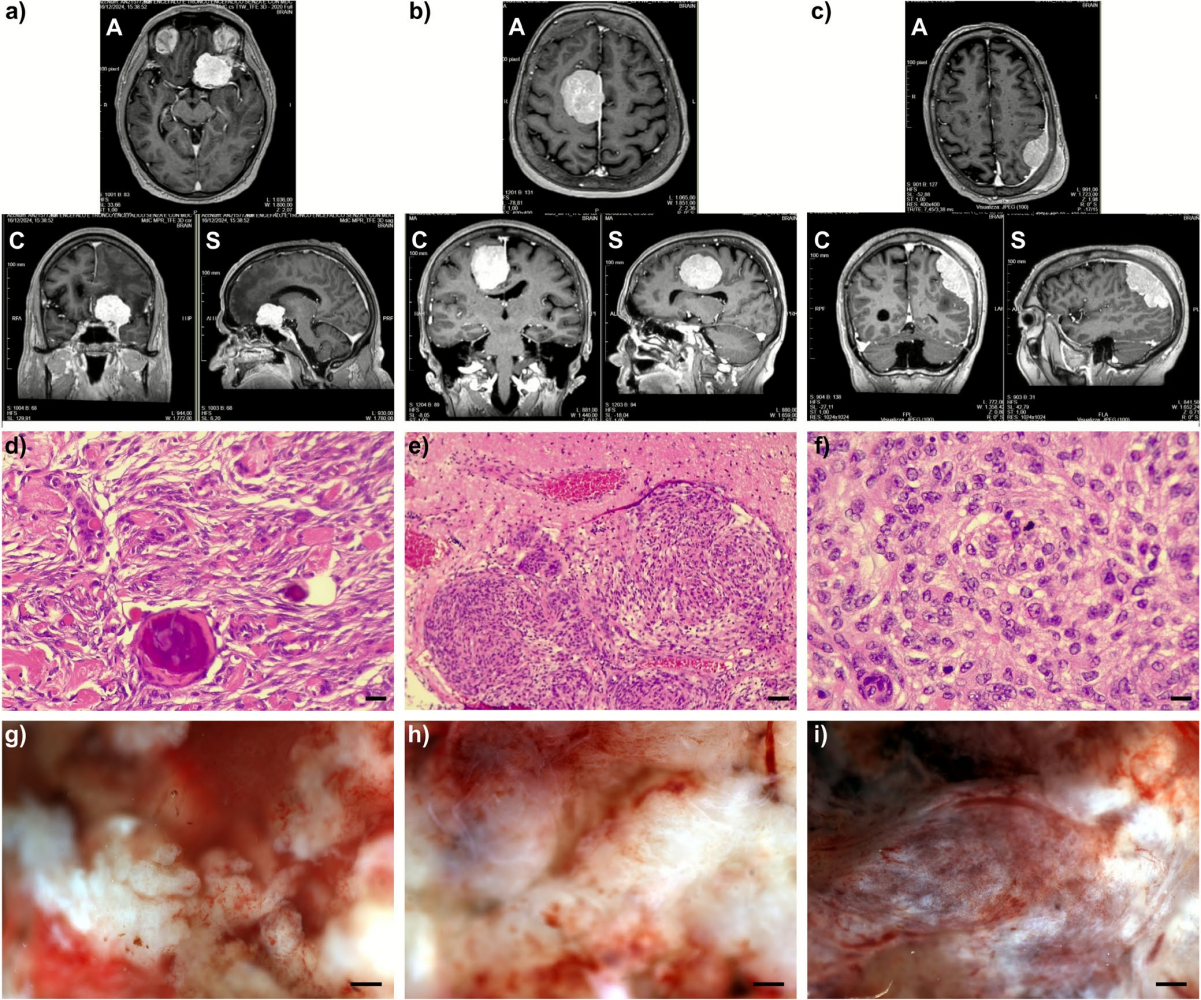
(a–c) Axial (A), coronal (C), and sagittal (S) projections of preoperative MRI (T1 with gadolinium) of a psammomatous meningioma, CNS WHO grade 1, an atypical meningioma, CNS WHO grade 2, and a anaplastic meningioma, CNS WHO grade 3, respectively; (d–f) Histological images of a psammomatous meningioma, an atypical meningioma, and an anaplastic meningioma, respectively, with hematoxylin and eosin (H&E); (d and f): Magnification 400×, scale bar 50 µm; (e): Magnification 200×, scale bar 100 µm. (g–i) RGB reconstruction from HSI imaging of a psammomatous meningioma, an atypical meningioma, and an anaplastic meningioma, scale bar 500 µm.

To illustrate the complementary information provided by different imaging modalities, Figure 2 presents three representative cases of meningioma of WHO grades 1, 2, and 3, arranged from left to right. The first row shows the corresponding preoperative MRI scans, the second row the postoperative histopathological sections (H&E‐stained), and the third row the RGB images reconstructed from hyperspectral acquisitions of the fresh tissue. This layout enables a direct visual comparison between radiological, histological, and optical information obtained from the same lesions.

The first case (left column) corresponds to a grade 1 transitional meningioma located at the left clinoid process, showing a compressive effect on the adjacent parenchyma with a 10 mm midline shift. The second case (middle column) depicts a grade 2 atypical meningioma anchored to the middle third of the cerebral falx, with moderate ventricular compression and areas of parenchymal infiltration. The third case (right column) illustrates a grade 3 anaplastic meningioma extending through the left parietal region with subcutaneous invasion. In each case, the postoperative H&E section confirms the histological grade based on the characteristic microscopic features of the subtype.

MRI provides detailed anatomical information, delineating lesion size, margins, and spatial relationships with surrounding structures. However, it offers limited biochemical specificity and cannot resolve the metabolic state of the tissue. Histology, which represents the gold standard for tumor grading, is primarily based on morphological criteria such as cellularity, mitotic activity, nuclear atypia, and the presence of necrosis. However, it is derived from fixed and stained sections that have undergone fixation, dehydration, paraffin embedding, and chemical processing. These steps remove lipids, denature proteins, and alter hemoglobin and cytochrome‐c oxidation states, precluding a physiologically meaningful comparison with biochemical measurements on fresh tissue. RGB images in the third row, obtained from acquired hypercubes of fresh resected samples, offer a complementary visualization, preserving the native optical and biochemical properties of the specimen. Although reconstructed from only three spectral bands, they already reveal distinct textures and coloration among tumors of different grades, reflecting differences in tissue structure and composition. Such qualitative contrast can support rapid identification of spatial patterns across the tissue surface, potentially aiding intraoperative decision‐making and highlighting regions of interest for further analysis. However, a deeper interpretation of these differences requires consideration of the entire hyperspectral cube, rather than the RGB composite alone. In the next section an analysis of such variability, an intrinsic and biologically relevant feature of meningiomas, is presented.

### 3.2 Molecular Distribution Analyses Through HyperProbe

**FIGURE 3:**
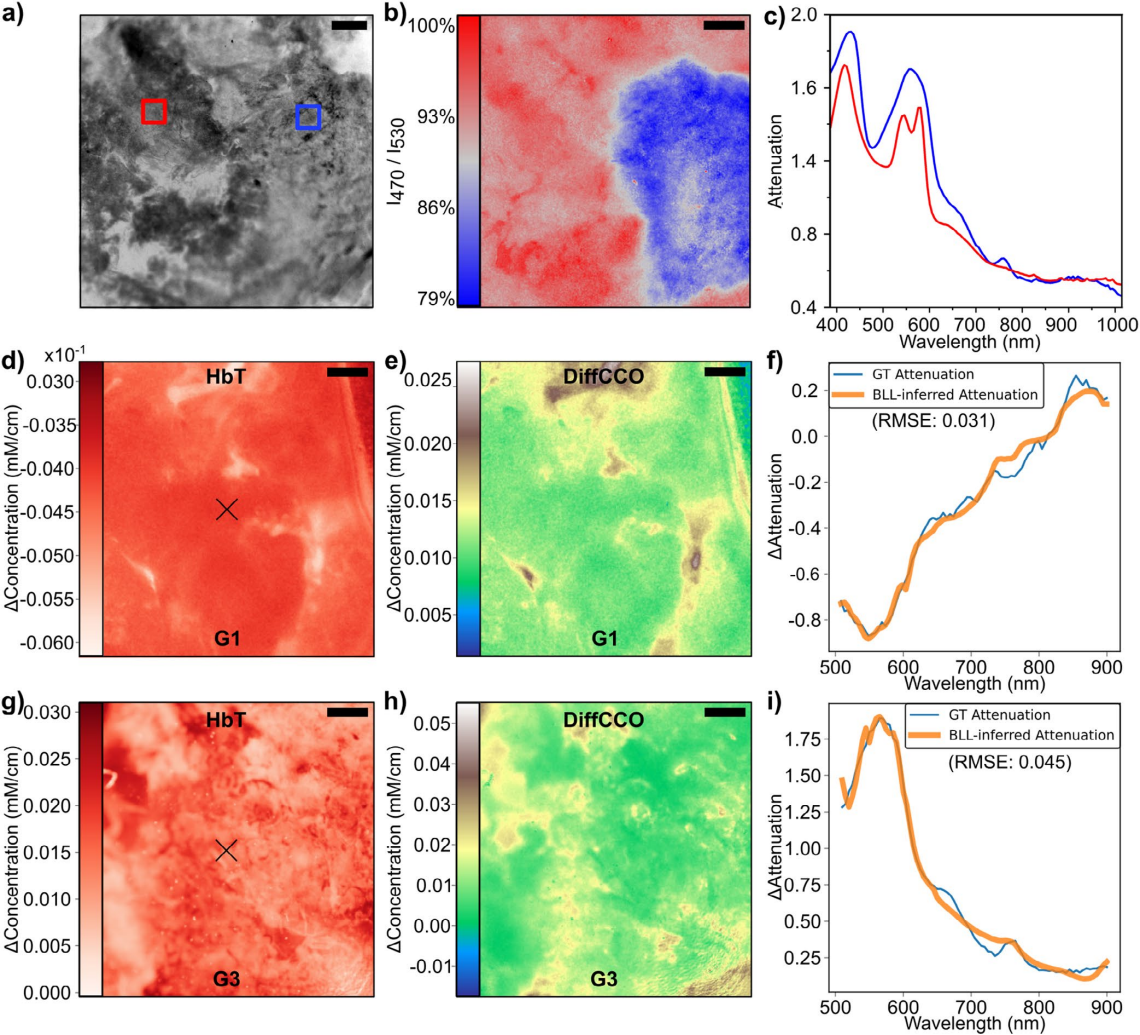
(a) A fresh meningioma biopsy's grayscale reflectance image illuminated with 400 nm light. Regions chosen for spectral analysis are shown by colored squares. Scale bar: 0.5 mm; (b) The oxygen saturation map is produced from the ratio of attenuation images at 470 and 530 nm. Scale bar: 0.5 mm; (c) Corresponding attenuation spectra from the red and blue regions in (a), demonstrating variations in the absorption characteristics of oxy‐ and deoxyhemoglobin; (d, e, g, h) Δ‐concentration maps of total hemoglobin (left) and differential cytochrome c oxidase (center) for a grade 1 (top row) and grade 3 (bottom row) meningioma. Values are expressed as deviations from the image mean. Scale bar: 0.5 mm; (f and i) GT attenuation spectra (blue lines) and corresponding Beer–Lambert Law (BLL)‐inferred fits (orange lines) for the pixel marked in the corresponding HbT map.

HP1.1 enables qualitative and quantitative content analysis, useful for meningioma characterization. Therefore, we first assessed the molecular composition of a meningioma through hyperspectral analysis, revealing intrasample variability in hemoglobin oxygenation states, not evident in conventional grayscale image (Figure 3a). In Figure 3c the attenuation spectra extracted from the selected regions (highlighted in red and blue in Figure 3a) reveal marked differences in the spectral profiles. Specifically, the graphed spectra show evident variations in the relative oxygenated and deoxygenated hemoglobin, with the characteristic absorbing features at 430–440 nm, 530–580 nm, and around 760 nm of HbO_2_ and HHb [19]. These spectral variations are most likely indicative of local tissue vascularisation, oxygenation, or tumor perfusion differences within the tumor mass. To visually highlight such a variation, in Figure 3b, we generated an oxygen saturation map of the original image presented in Figure 3a. In particular, we calculated the image attenuation ratio by dividing the image acquired at 470 nm, where HbO_2_ absorbs more strongly than HHb, by that at 530 nm, which lies near an isosbestic point and was used as a stable reference unaffected by oxygenation changes. Therefore, the ratio increases with higher oxygen saturation, enabling the generation of qualitative saturation maps that reflect spatial variations in tissue oxygenation. Figure 3d,e,g,h show inferred maps of concentration changes (Δ) for total hemoglobin (HbT) and DiffCCO in a grade 1 and a grade 3 meningioma, in the second and third row, respectively. The values represent deviations from the mean concentration across each image, emphasizing local variations within the tissue. In the rightmost subplots of each row (Figure 3f,i), we compare the ground‐truth (GT) attenuation spectra, and the corresponding model‐inferred attenuation obtained from the BLL at the selected pixel (marked with a black cross). The spectral agreement between GT and BLL curves reflects the model's ability to accurately infer underlying chromophore changes. The grade 3 meningioma exhibits a higher total hemoglobin concentration change and more heterogeneous DiffCCO distribution, indicative of increased vascularisation and metabolic activity. This is accompanied by a larger root mean square error (RMSE = 0.045), suggesting greater spectral complexity. In contrast, the grade 1 meningioma shows lower ΔHbT and ΔDiffCCO values, with an improved spectral fit (RMSE = 0.031), where the spectrum is slightly smoother and easier to fit. It indicates more surface homogeneity, consistent with a less aggressive tumor phenotype.

### 3.3 Inferred Hyperspectral Meningioma Classification

**FIGURE 4:**
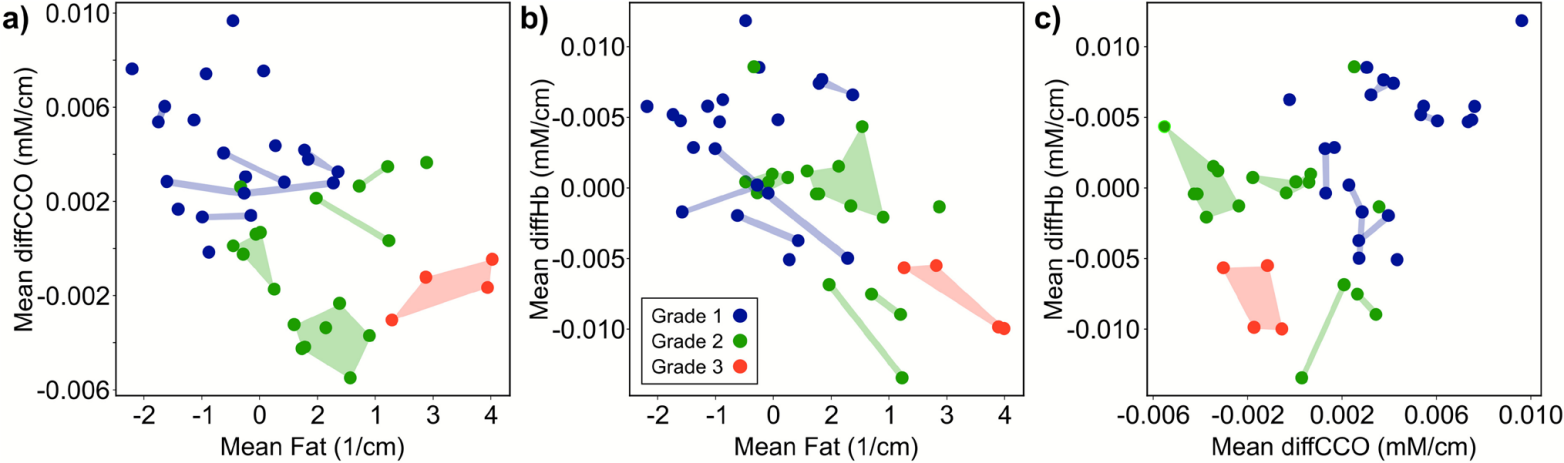
Spectral‐unmixing parameter plots showing qualitative trends across multiple fields of view (FOVs) from meningiomas of different grades. Each point corresponds to one FOV; colored shapes or connecting lines include values obtained from the same sample in adjacent FOVs; grade 1 (blue), grade 2 (green), and grade 3 (red). (a) Mean differential DiffCCO versus mean lipid (fat) content, (b) Mean DiffHb versus mean lipid content, (c) Mean differential hemoglobin (DiffHb) versus mean differential cytochrome c oxidase (DiffCCO).

Through spectral unmixing, we further investigate brain tumor contents such as fat (lipids), differential hemoglobin (DiffHb = oxyHb−deoxyHb), which reflect hemoglobin oxidation variations, and differential CCO (DiffCCO = oxCCO−redCCO), which indicate CCO concentration variations, revealing metabolic activity. Figure 4a–c illustrate the distributions of mean DiffCCO, DiffHb, and lipid content obtained from several FOVs across meningioma samples of different histological grades. Meningiomas are color‐coded by grade based on histopathological evaluation: grade 1 (G1) in blue, grade 2 (G2) in green, and grade 3 (G3) in red, respectively. Different points originating from the same specimen and acquired from adjacent FOVs, stand within the same colored shape (or connecting line), indicating that their molecular abundance values cluster within a narrow range. This confirms the internal coherence of the measurements within each sample. The slight dispersion observed among FOVs likely reflects genuine intratumoral heterogeneity in oxygenation and lipid metabolism rather than inconsistencies in histopathological grading, which provides a single label for the entire biopsy.

Although the small number of biological replicates prevents statistical inference, preliminary trends suggest grade‐related differences in lipid, hemoglobin and CCO signatures. G1 samples tend to group toward the upper left part of the plot of Figure 4a. These samples exhibit lower lipid content and increased levels of oxidized CCO (a key mitochondrial enzyme involved in oxidative metabolism). This profile may correspond to better‐perfused tissue with lower metabolic stress and minimal necrosis. The reduced lipid signature likely reflects a relatively preserved cellular environment with less membrane degradation or lipid accumulation, while the increased oxygenation suggests a viable microenvironment with good vascular function. G2 samples are concentrated in the central area of the plot and display intermediate or increased lipid content along with lower oxidized CCO compared to G1. This could indicate an environment with impaired oxygen delivery or increased consumption, associated with moderate hypoxia. The increase in the lipid component might be due to higher cell density, structural remodeling, or early degenerative processes. G3 samples lie in the lower‐right quadrant, showing the highest lipid content (fat values markedly larger than in other grades) and the lowest oxidized CCO signal. This pattern is compatible with highly aggressive and metabolically stressed tumors, often associated with extensive necrosis, lipid accumulation due to membrane breakdown, and compromised or chaotic vasculature resulting in oxygen deprivation. Figure 4b,c provide further insight into malignancy separation, highlighting the importance of CCO, DiffHb, and lipid as tumor biopsy biomarkers. Nevertheless, other combinations of molecules, including hemoglobin, manifest in greater overlap between malignancy clusters.

In summary, Figure 4 demonstrates that HSI combined with spectral unmixing is capable of extracting physiologically meaningful biomarkers, such as lipid burden and mitochondrial activity, that correspond closely with tumor grade. The observed clustering of tumor samples in the spectral feature space suggests that this method may serve as a rapid, label‐free optical biopsy, potentially supporting intraoperative decisions in real time. This supports the broader vision of the system as a comprehensive tool for metabolic and hemodynamic monitoring during neurosurgical procedures.

## 4 Discussion and Conclusions

This study demonstrates the potentialities of our HSI platform, the HP1.1, in the meningioma case scenario. The system is first of all able to retrieve both qualitative and quantitative information on the vascularity, perfusion, and metabolic heterogeneity of freshly resected brain tumor samples—parameters closely related to tumor viability and recurrence risk. Unlike single‐point spectroscopic probes, this system acquires the entire field simultaneously, preserving spatial information without mechanical scanning. This capability can be crucial for fast intraoperative assessment, tissue localization, and potential coregistration with histopathological sections.

Furthermore, the system proved the rapid assessment of endogenous biomarkers such as differential cytochrome c oxidase (DiffCCO), differential hemoglobin (DiffHb), and lipid content, providing biochemical signatures that reflect histopathological meningioma grades. Although the dataset is limited and slightly unbalanced (with only one grade 3 case) these preliminary results indicate that label‐free HSI can capture physiologically meaningful differences associated with tumor aggressiveness. Importantly, this study is conceived as a proof‐of‐concept investigation, aiming to evaluate the system's feasibility and potential diagnostic value in neurosurgical oncology. Due to the small number of biological replicates, we intentionally avoided inferential statistics or classifier development and restricted our analysis to qualitative trends. Nevertheless, the internal coherence of multiple FOVs per specimen supports the robustness of the measurements and highlights the system's ability to capture local heterogeneity.

Our technology also shows promise across a broader neuro‐oncological spectrum. Previous work with HP1.0 demonstrated its performance in gliomas [17], where subtle grade transitions often challenge intraoperative discrimination [20]. Extending this approach could further improve the differentiation of primary versus metastatic brain tumors [21], as variations in CCO activity and oxygenation reflect distinct metabolic phenotypes. For instance, specific spectral fingerprints from metastases originating from breast, lung, or melanoma primaries could inform tailored surgical strategies and enhance prognostic accuracy.

Future studies will expand the cohort and will include histopathological validation of tumor margins when ethically and surgically feasible. Notably, so far, HP1.1 has not been used intraoperatively in vivo, but only for ex vivo analysis of biological samples. Nevertheless, to validate these biomarkers under carefully monitored in vivo conditions, preclinical research in animal models—specifically, murine glioma or metastatic brain tumor models—will be essential. These investigations will also aid in evaluating how systemic physiological elements, like hypoxia or hyperoxia, affect hyperspectral measurements [14]. Translational research of this kind will ultimately facilitate the use of HSI in clinical settings and guide its integration into neuronavigation systems and surgical workflows. Hopefully, HP1.1 will work synergistically with the histological diagnosis that remains the gold standard.

## Funding

This work was supported by HORIZON EUROPE European Innovation Council and innovation program under grant agreement No 101071040—Project HyperProbe. I.T. from UCL is supported by the UK Research and Innovation (UKRI) (Grant No. 10048387). This research has also been supported by the Italian Ministry for University and Research in the framework of the Advanced Light Microscopy Italian Node of Euro‐Bioimaging ERIC.

## Conflicts of Interest

The authors declare no conflicts of interest.

## Data Availability

The data that support the findings of this study are available on request from the corresponding author. The data are not publicly available due to privacy or ethical restrictions.
